# Imaging of the Rare Association of Truncus Arteriosus in a Neonate with the Ductal Origin of the Left Subclavian Artery Using Multidetector CT Angiography and 3D Rendering

**DOI:** 10.7759/cureus.32131

**Published:** 2022-12-02

**Authors:** Saleh Alkathiri, Abby Walling, Dan L Russa, Ravi Ashwath, Sarv Priya

**Affiliations:** 1 Radiology, University of Iowa Hospitals and Clinics, Iowa City, USA; 2 Radiology, The Ottawa Hospital, Ottawa, CAN; 3 Pediatric Cardiology, University of Iowa Stead Family Children's Hospital, Iowa City, USA

**Keywords:** arch variation, 3d model, ct angiogram, isolated left subclavian artery, truncus arteriosus

## Abstract

We present the unreported case of a rare association of truncus arteriosus with the ductal origin of the left subclavian artery. Understanding and preoperative identification of these aortic variations are essential to guide optimal surgical management. In this study, the role of advanced visualization 3D modeling techniques in imaging these complex anomalies is discussed.

## Introduction

Truncus arteriosus (TA) is a rare congenital cardiac anomaly with a common trunk that gives rise to the aorta and the main pulmonary artery. TA can be associated with multiple vascular anomalies. An echocardiogram may be limited in the accurate identification of these anomalies. Complex anomalies can be accurately identified using cross-sectional imaging techniques, including computed tomography angiography (CTA) or magnetic resonance angiography (MRA). Advanced post-processing techniques, including volume rendering/3D modeling, may improve the understanding of these malformations.

## Case presentation

**Figure 1:**
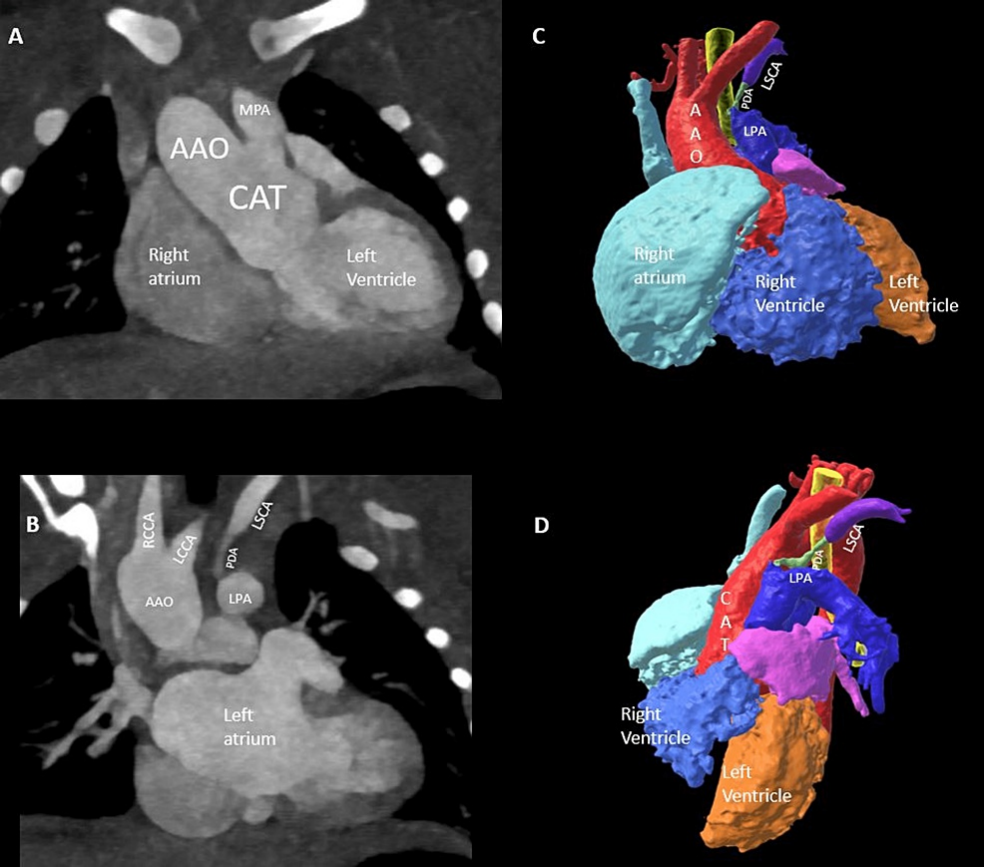
Truncus arteriosus with the ductal origin of the left subclavian artery. Coronal (A) reformatted CT angiogram image of the chest showing the type 1 truncus arteriosus. Reformatted coronal (B) CT image shows the origin of left subclavian artery (LSCA) from the ductus arteriosus (PDA), which, in turn, arises from the left pulmonary artery (LPA).  3D models frontal (C) and oblique (D) views show respective anatomy of truncus arteriosus with the ductal origin of left subclavian artery. CAT: Common arterial trunk, MPA: Main pulmonary artery, AAO: Ascending Aorta, LPA: Left pulmonary, artery, PDA: Patent ductus arteriosus, LSCA: Left subclavian artery, LCCA: Left common carotid artery, RCCA: Right common carotid artery.

A one-day-old female was born at 39 weeks of gestation with prenatal history of TA discovered on a fetal echocardiogram. Pregnancy was complicated by 22q11 deletion syndrome, polyhydramnios, and vanishing twin in the first trimester. The postnatal course was unremarkable, with an echocardiogram confirming the diagnosis of type 1 TA in the tricommissural truncal valve, a large subtruncal ventricular septal defect (VSD), and the normal origin of coronary arteries. The right aortic arch was suspected of aberrant origin of the left subclavian artery. A high-pitch gated CTA was performed to further evaluate for preoperative surgical anatomy (Siemens Drive, Erlangen Germany, 80kV, DLP 10 mGy.cm) after the administration of 7 ml of contrast at 1.5 ml/sec. The CTA showed a common arterial trunk giving rise to ascending aorta and the main pulmonary artery (Figure 1). The bifurcation of a short trunk of the main pulmonary artery into the right and left pulmonary arteries confirmed the diagnosis of type 1 TA (Figure 1). The right aortic arch was seen with the normal origin of the left common carotid, right common carotid, and right subclavian arteries (Figure 1). The left subclavian artery was seen to arise from the left ductus arteriosus (Figure 1).

There was a severe diffuse narrowing of ductus arteriosus arising from the left pulmonary artery with tight stenosis/occlusion at its ostial end (axial video).

**Video 1:** Axial video showing type 1 truncus and isolated ductal origin of the left subclavian artery.

Diffusely narrow left ductus arteriosus continued into normal caliber left subclavian artery (likely retrograde filling from a left vertebral artery). No aberrant origin of the left subclavian artery was seen. The neonate underwent surgical repair at 12 days of birth of TA, VSD closure, and right ventricle-pulmonary artery homograft conduit (pulmonary, 11 mm), and the ligation of the atretic ductus.

## Discussion

**Figure 2:**
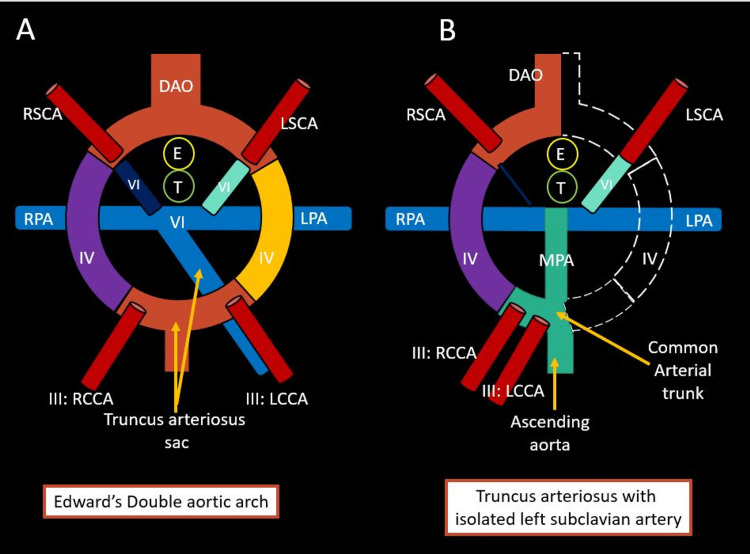
Edward’s double aortic arch model and truncus arteriosus with isolated subclavian artery. Figure 2A shows components of Edward’s hypothetical double aortic arch model. Third (III), fourth (IV), and sixth (VI) branchial arches are seen on both sides. Figure 2B shows the formation of TA with a common arterial trunk giving rise to ascending aorta and the main pulmonary artery. There is a persistence of the right aortic arch with involution of the left fourth aortic arch and left distal dorsal aorta (dashed lines) resulting in an isolated left subclavian artery (disconnected from the aortic arch). The left pulmonary artery is giving rise to the left subclavian artery via the left ductus arteriosus. The right ductus is obliterated. LCCA: Left common carotid artery; RCCA: Right common carotid artery; RSCA: Right subclavian artery; DAO: Unpaired fused dorsal aorta; LSCA: Left subclavian artery; RPA: Right pulmonary artery; LPA: Left pulmonary artery; T: Trachea; E: Esophagus.

TA is a rare cardiac malformation with a reported incidence rate of 2% [1]. Additionally, several associated aortic arch anomalies have been described, including the right aortic arch (most common), double aortic arch, ductus-dependent interrupted aortic arch, and aberrant right subclavian artery [2]. However, the ductal origin of the left subclavian artery (isolated) has yet to be described in patients with TA. Isolated left subclavian artery in patients with a right aortic arch is an extremely rare anomaly with an incidence rate of less than 1%. It is commonly associated with other congenital heart diseases [3]. Embryologically, the isolated left subclavian artery formation can be explained based on Edward’s double aortic arch model (Figure 2) [4].

The distal left dorsal aorta and left fourth arch involute (Figure 2), resulting in the isolation of the left subclavian artery from the aortic arch and left pulmonary artery, which gives rise to the left subclavian artery via ductus. In the case of ductal atresia, the left subclavian artery may be filled via ductus if it is patent or via the left vertebral artery or collaterals. Clinically, the isolated subclavian artery may be asymptomatic in neonates or present with pulmonary steal syndrome in the case of the patent ductus arteriosus. Alternatively, patients may present with upper limb pain due to hypoperfusion or subclavian steal syndrome. Surgical management in these patients involves correcting underlying cardiac defects and ligating the left ductus to prevent pulmonary steal syndrome. Antegrade restoration of flow to the isolated subclavian artery is controversial and depends on symptom severity. However, adequate collateral flow to the isolated subclavian artery must be ensured. Tetralogy of Fallot is the most commonly reported association with the right aortic arch and isolated subclavian artery. Other less common reported associated congenital heart diseases include VSD, d-transposition of great arteries, and bilateral ductii [3]. The association of the isolated left subclavian artery with TA, as seen in our case, has yet to be described.

## Conclusions

Complex cardiac malformations may have other associated vascular anomalies. Aortic arch variations have obvious surgical implications, and an echocardiogram may be limited in evaluating aortic arch branching pattern, as seen in our case. Thus, further imaging using low-dose CTA should be strongly considered for optimal preoperative surgical planning. Volume rendering images and 3D models may help better understand these complex vascular anomalies.
